# Prediction of improvement in skin fibrosis in diffuse cutaneous systemic sclerosis: a EUSTAR analysis

**DOI:** 10.1136/annrheumdis-2015-208024

**Published:** 2016-03-25

**Authors:** Rucsandra Dobrota, Britta Maurer, Nicole Graf, Suzana Jordan, Carina Mihai, Otylia Kowal-Bielecka, Yannick Allanore, Oliver Distler

**Affiliations:** 1 Division of Rheumatology, University Hospital Zurich, Zurich, Switzerland; 2 Department of Internal Medicine and Rheumatology, Cantacuzino Hospital, Carol Davila University of Medicine and Pharmacy, Bucharest, Romania; 3 Graf biostatistics, Winterthur, Switzerland; 4 Department of Rheumatology and Internal Medicine, Medical University of Bialystok, Bialystok, Poland; 5 Department of Rheumatology, University Paris Descartes and Cochin Hospital, Paris, France

**Keywords:** Systemic Sclerosis, Outcomes research, Epidemiology

## Abstract

**Objectives:**

Improvement of skin fibrosis is part of the natural course of diffuse cutaneous systemic sclerosis (dcSSc). Recognising those patients most likely to improve could help tailoring clinical management and cohort enrichment for clinical trials. In this study, we aimed to identify predictors for improvement of skin fibrosis in patients with dcSSc.

**Methods:**

We performed a longitudinal analysis of the European Scleroderma Trials And Research (EUSTAR) registry including patients with dcSSc, fulfilling American College of Rheumatology criteria, baseline modified Rodnan skin score (mRSS) ≥7 and follow-up mRSS at 12±2 months. The primary outcome was skin improvement (decrease in mRSS of >5 points and ≥25%) at 1 year follow-up. A respective increase in mRSS was considered progression. Candidate predictors for skin improvement were selected by expert opinion and logistic regression with bootstrap validation was applied.

**Results:**

From the 919 patients included, 218 (24%) improved and 95 (10%) progressed. Eleven candidate predictors for skin improvement were analysed. The final model identified high baseline mRSS and absence of tendon friction rubs as independent predictors of skin improvement. The baseline mRSS was the strongest predictor of skin improvement, independent of disease duration. An upper threshold between 18 and 25 performed best in enriching for progressors over regressors.

**Conclusions:**

Patients with advanced skin fibrosis at baseline and absence of tendon friction rubs are more likely to regress in the next year than patients with milder skin fibrosis. These evidence-based data can be implemented in clinical trial design to minimise the inclusion of patients who would regress under standard of care.

## Introduction

Systemic sclerosis (SSc) is a highly heterogeneous disease, making clinical management of SSc and patient selection for clinical trials challenging. For skin fibrosis, the modified Rodnan skin score (mRSS) is the most widely used measure in clinical practice and it is also the most frequent primary end point in clinical trials.^1^ ^2^ Identifying predictors of change in mRSS over time is therefore of much interest for risk-tailored clinical management, as well as for clinical trial design to enrich for patients with worsening skin fibrosis.^3^

In a recent, large-scale, observational study on the European Scleroderma Trials And Research (EUSTAR) database, we identified short disease duration (<15 months), joint synovitis and low baseline mRSS (>7/51 and ≤22/51) as independent predictors of skin progression in patients with diffuse cutaneous SSc (dcSSc).^4^ This provided an evidence-based tool for the improved identification of patients at risk for progressive skin involvement and is also valuable for cohort enrichment in clinical trials on skin fibrosis.

While the identification of factors predicting progression has been improved, little is known about predictors of regression of skin fibrosis in patients with SSc. Regression of mRSS has long been identified as a characteristic feature of the natural history of skin fibrosis in patients with dcSSc. Regression is thought to occur after the early active phase of the disease has stabilised. However, the time to peak skin score varies widely in patients, leading to a highly heterogeneous pattern of regressing, progressing and stable patients even in early disease stages.^5^ ^6^

From a therapeutic perspective, there is general agreement that prevention of progression in patients with active skin fibrosis is one of the major treatment goals. However, it is much less established whether a therapeutic benefit can be achieved for patients who are already likely to show improvement of skin fibrosis under standard of care. In such a population the benefit/risk ratio of any treatment would have to be accurately assessed. Therefore, in order to identify those patients who would benefit most from therapeutic interventions, it is important to identify patients with progressive skin fibrosis, and to be aware of patients with skin regression. For trial design, it is important to enrich for progressive patients, and to exclude regressing patients under standard of care to increase the likelihood of identifying treatment effects.

So far, previous attempts to identify predictors of change in mRSS have been largely inconclusive,^4^ and patients recruited for clinical studies targeting skin fibrosis often show spontaneous regression of mRSS.^4^ ^5^ Thus, the objective of our study was to provide evidence-based predictors of skin improvement in patients with dcSSc using the EUSTAR database.

## Methods

### Patients and study design

The longitudinally followed EUSTAR cohort was analysed for this observational study. The whole EUSTAR data set, consisting of 12 274 patients at the time of the first data export (20 February 2015), was considered.

The following inclusion criteria were used for cohort selection: fulfilment of American College of Rheumatology 1980 classification criteria, dcSSc, mRSS ≥7 at the first visit (baseline) and available data for mRSS at 12±2 months follow-up.

Patients with dcSSc were identified according to LeRoy *et al*^7^ or, in case of missing values for the LeRoy criteria, by diffuse skin involvement at any visit. The minimum mRSS ≥7 was chosen because it reflects the lowest value classifiable as dcSSc, thus allowing the inclusion of patients with dcSSc with less severe to extensive skin fibrosis. The 1 year follow-up has been shown adequate for capturing significant changes in mRSS and is often used in clinical trials in skin fibrosis in SSc.^2^

The clinical data in EUSTAR are prospectively collected in a multicentre approach following a standardised protocol.^8–10^ Regular training courses in skin scoring are organised by EUSTAR and all centres are advised to have the same examiner assessing the skin score in individual patients at follow-up visits.^4^ Quality indicators for data from the registry include regular external monitoring of large centres and plausibility checks on key items with written requests to centres for clarification. Ethics approval has been obtained from the respective local ethics committees by all participating EUSTAR centres.

### Statistical analysis

The primary end point, improvement of skin fibrosis, was defined as a decrease in mRSS of >5 points and ≥25% within 1 year. These thresholds were chosen according to the minimal clinically important difference.^11^ Similarly, progression of skin fibrosis was defined as an increase in mRSS of >5 points and ≥25% within 1 year as used previously.^4^ For definitions of the clinical variables see the online supplement.

10.1136/annrheumdis-2015-208024.supp1 Supplementary data

A subanalysis using receiver operating characteristic analysis was performed with skin improvement, and, respectively, skin progression as the state variable, in order to explore the relationship between different baseline mRSS cut-off points and the proportion of regressors and progressors included in the cohort.

Candidate predictors for skin improvement were selected based on nominal group technique by SSc experts (OD, YA, OK-B, CM, RD), who were asked to suggest clinically meaningful variables with face validity (see the online supplement). All parameters suggested by the experts (see online supplementary table S1) with <50% missing data were considered for the analysis. As a first step, a multivariable logistic regression model including all selected 11 parameters was run after single conditional mean imputation of the data. Baseline mRSS was centred at 7 points as all patients had baseline mRSS ≥7. Because baseline mRSS did not behave linearly, a quadratic term for baseline mRSS was included in the model. The Wald statistics (see online supplementary table S3) showed that the effects were very far from being significant (p value >0.7) for joint contractures and diffusing capacity of the lung for carbon monoxide ≥70%. Thus, these parameters were excluded from further models. The interaction between disease duration and baseline mRSS was also tested, but proved to be insignificant, meaning that the effect of baseline mRSS on regression of SSc did not depend on disease duration. The model after single imputation is shown in the online supplementary table S4. Single imputation was done for validation as it is not possible to validate models with multiply imputed data. Thus, bootstrap with 100 repetitions was used to validate the model (see online supplementary table S5). However, as multiple imputation provides more trustworthy estimates and ORs than single imputation, the final logistic regression model from the multiply imputed data set is presented.

The statistical analysis was performed by the biostatistician (NG) using R V.3.1.0 (see the online supplement).

## Results

### Study population

A total of 919 patients with dcSSc who met the inclusion criteria were analysed. Of these, 218/919 (24%) patients showed skin improvement over 1-year follow-up. The patients' demographic and clinical characteristics are shown in table 1.

**Table 1:** Description of the study cohort at baseline (total N=919)

Age (years)	51 _(__40,60)_
Male	199/919 (21.7)
Female	720/919 (78.3)
Disease duration (months)*	42.5 _(17,104)_
Short disease duration ≤36 months*	389/854 (45.6)
Raynaud's phenomenon	898/919 (97.7)
Puffy fingers	235/390 (60.3)
Digital ulcers (ever)	396/914 (43.3)
Active digital ulcers	84/394 (21.3)
mRSS	16 _(11,23)_
*Organ involvement*	
Musculoskeletal	
Synovitis†	196/915 (21.4)
Joint contractures	458/916 (50.0)
Tendon friction rubs	185/914 (20.2)
Muscle weakness	294/915 (32.1)
*Cardiopulmonary*	
Dyspnoea (NYHA)	
Stage I	210/369 (56.9)
Stage II	118/369 (32.0)
Stage III	37/369 (10.0)
Stage IV	4/369 (1.1)
Conduction blocks	109/877 (12.4)
Diastolic dysfunction	165/864 (19.1)
LVEF <45%	6/318 (1.9)
Pulmonary hypertension by Echo	167/868 (19.2)
Lung fibrosis on chest X-ray	389/849 (45.8)
Lung fibrosis on HRCT	163/287 (56.8)
FVC <80%	127/362 (35.1)
TLC <80%	80/245 (32.7)
DLCO <70%	357/621 (57.5)
*Gastrointestinal*	
Oesophageal symptoms	625/917 (68.2)
Stomach symptoms	257/916 (28.1)
Intestinal symptoms	222/917 (24.2)
*Renal crisis*	23/915 (2.5)
Laboratory markers	
ANA	859/908 (94.6)
ACA	76/872 (8.7)
Anti-Scl70	524/886 (59.1)
Anti-U1RNP	16/285 (5.6)
Anti-RNA polymerase III	18/215 (8.4)
CK elevation	112/879 (12.7)
Proteinuria	74/887 (8.3)
ESR>25 mm/1 h	134/368 (36.4)
CRP elevation	107/374 (28.6)
Active disease (VAI >3)^12^	146/337 (43.3)
Immunosuppressive treatment	334/436 (76.6)

For nominal variables, the absolute and relative frequencies are shown: n/total valid cases (%). Continuous variables are described as median and 1st, 3rd quartiles (Q1, Q3).

*Disease duration was calculated as difference between the date of the baseline visit and the date of the first non-Raynaud's symptom of the disease, as reported by the patients.

†Joint synovitis was defined as swelling of the joints as judged by the treating physician.

ACA, anticentromere antibodies; ANA, antinuclear antibodies; Anti-Scl70 antibodies, antitopoisomerase I antibodies; CK, creatine kinase; CRP, C reactive protein; DLCO, diffusing capacity of the lung for carbon monoxide; Echo, echocardiography; ESR, erythrocyte sedimentation rate; FVC, forced vital capacity; HRCT, high resolution computer tomography; LVEF, left ventricular ejection fraction; mRSS, modified Rodnan skin score; NYHA, New York Heart Association; RNP, ribonucleoprotein; TLC, total lung capacity; VAI, Valentini Activity Index.

### Multivariable analysis

The candidate predictors selected after nominal group technique and exclusion of parameters with higher missing values and finally included in the multivariable analysis are shown in box 1.

Box 1 **Candidate predictors of skin improvement selected for the analysis**

Variable

- Baseline mRSS
- Disease duration
- ANA positive
- Anti-Scl70 positive
- Tendon friction rubs
- Proteinuria
- Conduction blocks
- Abnormal diastolic function
- Fibrosis on chest X-ray
- DLCO ≥70%
- Joint contractures

ANA, antinuclear antibodies; Anti-Scl70 antibodies, antitopoisomerase I antibodies; DLCO, diffusing capacity of the lung for carbon monoxide; mRSS, modified Rodnan skin score.

The prediction model for skin improvement after single imputation is shown in online supplementary table S4. The most significant predictor was baseline mRSS (p<0.001). Other significant predictors were absence of tendon friction rubs and negative Scl-70 antibodies. The performance parameters of the model before and after validation are shown in online supplementary table S5.

Multiple imputation was used to fit the model and to obtain SEs. The final model with multiply imputed data is shown in table 2.

**Table 2:** Final logistic regression model for prediction of skin improvement at 1 year

Predictors	Estimate	SE	OR	p Value	95% CI
ANA positive	−0.378	0.339	0.685	0.266	0.352 to 1.334
Anti-Scl70 positive	−0.324	0.180	0.723	0.071	0.508 to 1.028
Tendon friction rubs	−0.492	0.214	0.612	0.022	0.402 to 0.930
Proteinuria	0.294	0.292	1.341	0.315	0.756 to 2.380
Lung fibrosis	0.112	0.179	1.119	0.530	0.787 to 1.590
Disease duration (months)	−0.001	0.001	0.999	0.287	0.997 to 1.001
Baseline mRSS	0.171	0.031	1.186	<0.001	1.115 to 1.262
Baseline mRSS^2^	−0.003	0.001	0.997	0.004	0.996 to 0.999
Intercept	−3.233	0.538	0.039	<0.001	0.014 to 0.113

ANA, antinuclear antibodies; Anti-Scl70 antibodies, antitopoisomerase I antibodies; mRSS, modified Rodnan skin score.

High baseline mRSS remained the strongest predictor of skin improvement (p<0.001). The model for example indicates that the risk for regression within 12 months is more than doubled (OR=2.316) for a patient with a baseline mRSS of 22 points, in comparison to a patient with a baseline mRSS of 14 (all other parameters being equal). Furthermore, absence of tendon friction rubs significantly predicted skin improvement. Absence of anti-Scl-70 antibodies, which was also a significant predictor in the model after single imputation (see online supplementary table S3), only retained a trend in the model from the multiply imputed data set.

### Baseline mRSS as predictor of the pattern of skin change in dcSSc over 1 year

The observation that high baseline mRSS was the strongest predictor of skin improvement complements our previous findings indicating low baseline mRSS as an important predictor of skin worsening.^4^ This was also confirmed in the current cohort: the 95/919 (10%) patients with dcSSc who showed skin progression within 1 year had lower baseline mRSS (p<0.001). Baseline mRSS is thus a predictor of change in skin score after 1 year, patients with lower skin scores being prone to progress and those with higher skin scores prone to improve within the next 12 months (see online supplementary figure S1).

**Figure 1:**
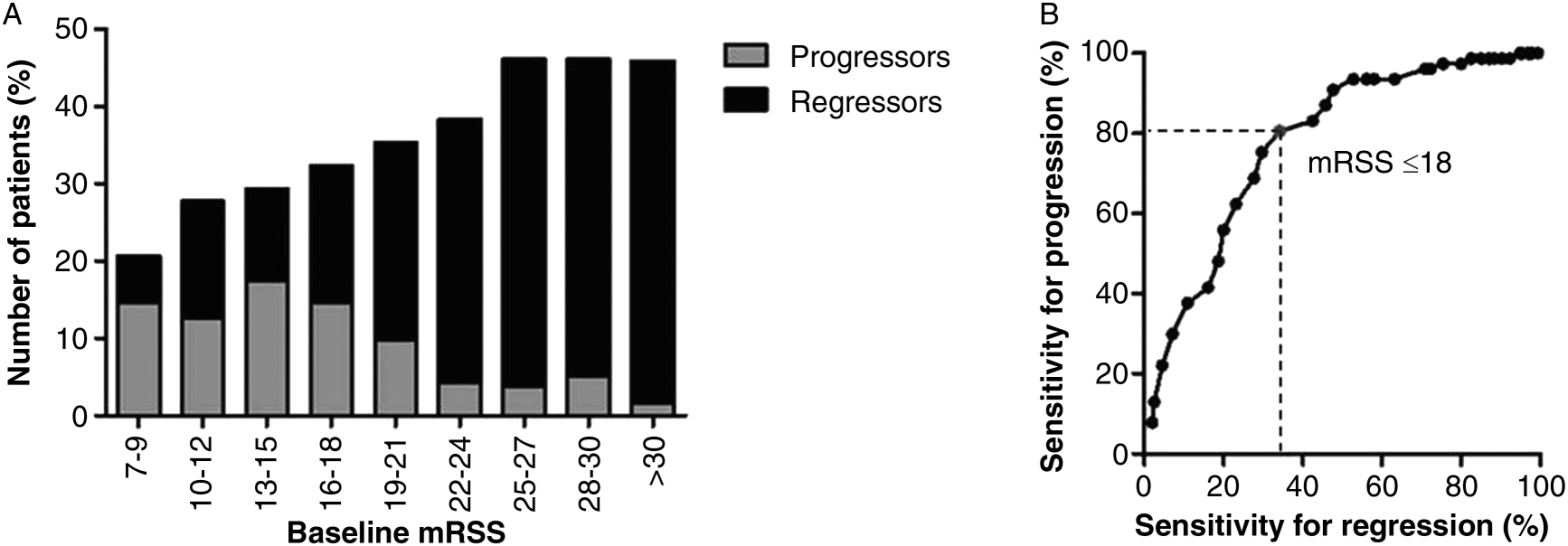
(A) Percentage of progressors and regressors per baseline modified Rodnan skin score (mRSS) range. Patients with lower skin score are more likely to progress, whereas those with higher skin scores are much more likely to regress. (B) Sensitivity for progression and regression depending on different cut-off values for baseline mRSS.

Having in mind the optimisation of cohort enrichment with maximal number of progressive patients and minimal numbers of regressive patients, we checked for the optimal mRSS cut-off (figure 1). In this cohort, an upper baseline mRSS cut-off value of 18 points performed best, including the highest proportion of progressors (78.9%) and the lowest proportion of regressors (35.3%, figure 1).

If translated into clinical study design, this suggests a baseline mRSS between 7 and 18 as an inclusion criterion, raising questions for feasibility of recruiting patients. Thus, we next analysed the impact of higher cut-offs for upper baseline mRSS on the proportion of progressive and regressive patients. This analysis showed that a baseline mRSS between 18 and 25 would still allow identifying a reasonably high rate of progressors over regressors, whereas for skin scores higher than 25 a considerable drop of the included progressors and a dramatic increase in the percentage of regressors was observed (table 3).

**Table 3:** Proportion of progressors and regressors depending on different cut-offs for baseline mRSS

mRSS cut-off	Progressors (%)	95% CI*	Regressors (%)	95% CI*
<18	13.70	10.99 to 16.95	12.92	10.28 to 16.10
<19	13.64	11.02 to 16.76	14.00	11.35 to 17.15
<20	13.15	10.67 to 16.11	15.51	12.82 to 18.65
<21	13.20	10.77 to 16.07	16.06	13.40 to 13.13
<22	13.11	10.74 to 15.91	16.62	13.96 to 19.66
<23	12.77	10.48 to 15.47	17.42	14.77 to 20.43
<24	12.68	10.43 to 15.33	17.89	15.24 to 20.88
<25	12.38	10.18 to 14.97	18.57	15.91 to 21.56
≤25	11.94	9.81 to 14.45	19.23	16.58 to 22.20
>25	3.03	1.30 to 6.90	44.24	36.88 to 51.87

Data are shown as percentage (%) from the total number of patients (N=919).

*Newcombe–Wilson method without continuity correction.

mRSS, modified Rodnan skin score.

## Discussion

Patients with improvement of skin fibrosis under standard of care are less likely to benefit from therapeutic interventions than patients with progressive skin fibrosis. In this large EUSTAR analysis of 919 patients with dcSSc with clinically derived real life data, we have identified parameters which can predict improvement of skin fibrosis over a 12 month observation period, the strongest being high baseline mRSS.

To our knowledge, this is the first report of an evidence-based model for the prediction of skin improvement in a non-selected cohort of patients with dcSSc. In a previous study, Steen *et al* specifically focused on improvement of skin thickening in a cohort of 278 patients with early dcSSc. In this study, independent predictors for skin improvement could not be identified (except for D-Penicillamine use, which is however contradictory to the negative results of the dedicated randomised controlled trial).^13–15^ Potential explanations for this lack of predictors include the lower sample size, and the higher baseline mRSS in this study. The higher baseline mRSS might also explain the higher rate of improvers in this study (63% vs 22% in our EUSTAR analysis).

A key message resulting from our study is the important role of baseline mRSS to predict either progression or regression of skin fibrosis. This also supports our previous EUSTAR analysis on worsening of skin fibrosis.^4^ In a pooled analysis of patients with dcSSc from seven multicentre clinical trials, mRSS at baseline had a weak negative correlation with any change in mRSS.^6^ Furthermore, in a recent analysis from the Canadian cohort, baseline mRSS was the only baseline parameter significantly associated with a significant change in mRSS at follow-up (defined as difference in 8 points).^16^ These data underline the value of mRSS for cohort enrichment in clinical trials. Our study also provides evidence-derived data on different thresholds of baseline mRSS and their performance to enrich for progressors over regressors in clinical trials (table 3). Thus, the optimal baseline mRSS cut-off for a specific study can be chosen from these data taking into account feasibility versus optimised cohort enrichment.

Another important aspect to consider is the natural regression to the mean phenomenon: the more extreme the skin score values in the study population at baseline, the more likely they are to decrease towards the mean at follow-up, thus not necessarily reflecting treatment response. The regression to the mean is most likely the statistical effect that explains the selection of baseline mRSS as a strong predictor of worsening and regression, respectively.

While our study addresses a very large SSc cohort with multiple data quality controls and external data monitoring, it also has several limitations. It has the natural drawbacks of registry data, such as missing data. However, we have addressed this issue by applying acknowledged imputation methods to compensate for missing data. Nonetheless, some of the candidate predictors had too much missing data which did not allow a trustable imputation, hence we could not include them in the analysis. The inclusion threshold of mRSS>7 aimed at identifying patients with true dcSSc with minimum involvement of distal upper extremities, but is, nonetheless, somewhat arbitrary. Our data also have to be confirmed in other cohorts with different baseline characteristics, for example, with higher prevalence of anti-RNA polymerase III antibodies. Additional information from in-between visits (eg, at 3 months, 6 months) as well as health assessment questionnaire data^17^ could bring additional valuable information on the course of skin fibrosis. Moreover, it has to be mentioned that the final prediction model only explains about 16% of the variation in skin fibrosis regression (see online supplementary table S4), indicating that other yet unknown factors such as, for example, biomarkers have an important role in determining the improvement of skin fibrosis in dcSSc. Further, modifications on how to measure the mRSS (eg, averaging vs maximising skin thickness over a certain skin area) will have great impact on the specific baseline mRSS values and have to be considered when these data are applied to clinical trials.

In conclusion, our study provides novel evidence-based data for cohort enrichment in clinical trials on skin fibrosis in patients with dcSSc. These data further support a lower baseline mRSS as inclusion criteria to optimise the ratio of progressors over regressors for recruitment into clinical trials. Other significant predictors of improvement with potential application to clinical trials resulting from these data are absence of tendon friction rubs, and, potentially, negativity for anti-Scl70 antibodies.
