# Supplementary material for: Prediction of improvement in skin fibrosis in diffuse cutaneous systemic sclerosis: a EUSTAR analysis
Source: Ann Rheum Dis. 2016 Mar 25;75(10):1743–8. doi: 10.1136/annrheumdis-2015-208024 (PMC5036205; doi:10.1136/annrheumdis-2015-208024)
Supplement: Supplementary data [file annrheumdis-2015-208024supp.pdf]

Supplement

Prediction of Improvement in Skin Fibrosis in Diffuse Cutaneous Systemic Sclerosis – a EUSTAR Analysis

Rucsandra Dobrota[1,2], Britta Maurer[1], Nicole Graf[3], Carina Mihai[2], Suzana Jordan[1], Otylia Kowal-Bielecka[4], Yannick Allanore[5], Oliver Distler[1] on behalf of EUSTAR co-authors

Collaborators of the EUSTAR centres (numerical order of centers)

Marco Matucci Cerinic[6], Serena Guiducci[6], Ulrich Walker[7], Giovanni Lapadula[8], Florenzo Iannone[8], Radim Becvar[9], Stanislaw Sierakowsky[4], Maurizio Cutolo[10], Alberto Sulli[10], Gabriele Valentini[11], Giovanna Cuomo[11], Serena Vettori[11], Gabriela Riemekasten[12], Elise Siegert[13], Simona Rednic[14], Ileana Nicoara[14], André Kahan[5], P. Vlachoyiannopoulos[15], C. Montecucco[16], Roberto Caporali[16], Patricia E. Carreira[17], Srdan Novak[18], László Czirják[19],Cecilia Varju[19], Carlo Chizzolini[20], Eugene J. Kucharz[21], Anna Kotulska[21], Magdalena Kopec-Medrek[21], Malgorzata Widuchowska[21], Franco Cozzi[22], Blaz Rozman[23], Carmel Mallia[24], Bernard Coleiro[24], Armando Gabrielli[25], Dominique Farge[26], Chen Wu[26], Zora Marjanovic[26], Helene Faivre[26], Darin Hij[26], Roza Dhamadi[26], Paolo Airò[27], Roger Hesselstrand[28], Frank Wollheim[28], Dirk M Wuttge[28], Kristofer Andréasson[28], Duska Martinovic[29], Alexandra Balbir-Gurman[30], Yolanda Braun-Moscovici[30], F. Trotta[31], Andrea Lo Monaco[31], Nicolas Hunzelmann[32], Raffaele Pellerito[33], Lisa Maria Bambara[34], Paola Caramaschi[34], Carol Black[35], Christopher Denton[35], Nemanja Damjanov[36], Jörg Henes[37], Vera Ortiz Santamaria[38], Stefan Heitmann[39], Dorota Krasowska[40], Matthias Seidel[41], Harald Burkhardt[42], Andrea Himsel[42], Maria J. Salvador[43], José Antonio Pereira Da Silva[43], Bojana Stamenkovic[44], Aleksandra Stankovic[44], Mohammed Tikly[45], Lidia P. Ananieva[46], Lev N. Denisov[46], Ulf Müller-Ladner[47], Marc Frerix[47], Ingo Tarner[47], Raffaella Scorza[48], Merete

Engelhart[49], Gitte Strauss[49], Henrik Nielsen[49], Kirsten Damgaard[49], Antonio Zea Mendoza[50], Carlos de la Puente[50], Walter A. Sifuentes Giraldo[50], Øyvind Midtvedt[51], Silje Reiseter[51], Eric Hachulla[52], David Launay[52], Guido Valesini[53], Valeria Riccieri[53], Ruxandra Maria Ionescu[54], Daniela Opris[54], Laura Groseanu[54], Roxana Sfrent Cornateanu[2], Razvan Ionitescu[2], Ana Maria Gherghe[2], Alina Soare[2], Marilena Gorga[2], Mihai Bojinca[2], Georg Schett[55], Jörg HW Distler[55], Christian Beyer[55], Pierluigi Meroni[56], Francesca Ingegnoli [56], Luc Mouthon[57], Filip De Keyser[58], Vanessa Smith[58], Francesco P. Cantatore[59], Ada Corrado[59], Maria R. Pozzi[60], Kilian Eyerich[61], Rüdiger Hein[61], Elisabeth Knott[61], Piotr Wiland[62], Magdalena Szmyrka-Kaczmarek[62], Renata Sokolik[62], Ewa Morgiel[62], Marta Madej[62], Brigitte Krummel-Lorenz[63], Petra Saar[63], Martin Aringer[64], Claudia Günther[64], Rene Westhovens[65], Ellen de Langhe[65], Jan Lenaerts[65], Branimir Anic[66], Marko Baresic[66], Miroslav Mayer[66], Sebastião C. Radominski[67], Carolina de Souza Müller[67], Valderílio F. Azevedo[67], Svetlana Agachi[68], Liliana Groppa[68], Lealea Chiaburu[68], Eugen Russu[68], Sergei Popa[68], Thierry Zenone[69], Simon Stebbings[70], John Highton[70], Lisa Stamp[71], Peter Chapman[71], John O'Donnell[71], Kamal Solanki[72], Alan Doube[72], Douglas Veale[73], Marie O'Rourke[73], Esthela Loyo[74], Mengtao Li[75], Edoardo Rosato[76], Antonio Amoroso[76], Antonietta Gigante[76], Cristina-Mihaela Tanaseanu[77], Monica Popescu[77], Alina Dumitrascu[77], Isabela Tiglea[77], Rosario Foti[78], Rodica Chirieac[79], Codrina Ancuta[79], Peter Villiger[80], Sabine Adler[80], Paloma García de la Peña Lefebvre[81], Silvia Rodriguez Rubio[81], Marta Valero Exposito[81], Jean Sibilia[82], Emmanuel Chatelus[82], Jacques Eric Gottenberg[82], Hélène Chifflot[82], Ira Litinsky[83], Algirdas Venalis[84], Irena Butrimiene[84], Paulius Venalis[84], Rita Rugiene[84], Diana Karpec[84], Lesley Ann Saketkoo[85], Joseph A. Lasky[85], Eduardo Kerzberg[86], Fabiana Montoya[86], Vanesa Cosentino[86], Massimiliano Limonta[87], Antonio Luca Brucato[87], Elide Lupi[87], François Spertini[88], Camillo Ribi[88], Guillaume Buss[88], Jean Louis Pasquali[89], Thierry Martin[89], Audrey Gorse[89]

[1]Division of Rheumatology, University Hospital Zurich, Zurich, Switzerland

[2]Department of Internal Medicine and Rheumatology, Cantacuzino Hospital, Carol Davila University of Medicine and Pharmacy, Bucharest, Romania

[3]graf biostatistics, Winterthur, Switzerland

[4]Department of Rheumatology and Internal Medicine, Medical University of Bialystok, Bialystok, Poland

[5]Department of Rheumatology, University Paris Descartes and Cochin Hospital, Paris, France

[6]Department of Medicine, Section of Rheumatology, University of Florence, Italy

[7]Department of Rheumatology, University Hospital Basel, Switzerland

[8]Rheumatology Unit-DiMIMP, School of Medicine University of Bari, Italy

[9]Institute of Rheumatology, 1st Medical School, Charles University, Prague, Czech Republic

[10]Research Laboratory and Division of Rheumatology, Department of Internal Medicine, University of Genova, Italy

[11]Department of Clinical and Experimental Medicine “F-Magrassi” II Policlinico, Unit of Rheumatology, Naples, Italy

[12]Department of Rheumatology, University of Lübeck, Germany

[13]Department of Rheumatology, Charitè University Hospital, Berlin, German Rheumatism Research Centre Berlin (DRFZ), a Leibniz institute, Germany

[14]Department of Rheumatology, University of Medicine & Pharmacy "Iuliu Hatieganu" Cluj, Cluj-Napoca, Romania

[15]Department of Pathopysiology, Medical School, National University of Athens, Greece

[16]Unita' Operativa e Cattedra di Reumatologia, IRCCS Policlinico S Matteo, Pavia, Italy

[17]Division of Rheumatology, Hospital 12 de Octubre, Madrid, Spain

[18]Department of Rheumatology and Clinical Immunology, Internal Medicine, KBC Rijeka, Croatia

[19]Depatment of Immunology and Rheumatology, Faculty of Medicine, University of Pécs, Hungary

[20]Department of Immunology and Allergy, University Hospital, Geneva, Switzerland

[21]Department of Internal Medicine and Rheumatology, Medical University of Silesia,Katowice, Poland

[22]Rheumatology Unit, Department of Clinical and Experimental Medicine, Universityof Padova, Italy

[23]University Medical Center Ljublijana, Division of Internal Medicine, Department of Rheumatology, Ljubliana, Slovenia

[24]"Stella Maris", Balzan, Malta

[25]Dipartimento di Scienze Cliniche e Molecolari, Clinica Medica, Università Politecnica delle Marche, Ancona, Italy

[26]Department of Internal Medicine, Hospital Saint-Louis, Paris, France

[27]Spedali Civili di Brescia, Servizio di Reumatologia Allergologia e Immunologia Clinica, Brescia, Italy

[28]Department of Rheumatology, Lund University, Lund, Sweden

[29]Department of Internal Medicine, Clinical Hospital of Split, Croatia

[30]B. Shine Rheumatology Unit, Rambam Health Care Campus, Rappaport Faculty of Medicine, Technion, Haifa, Israel

[31]Department of Clinical and Experimental Medicine, Rheumatology Unit, University of Ferrara, Italy

[32]Department of Dermatology, University Hospital Cologne, Germany

[33]Ospedale Mauriziano, Centro di Reumatologia, Torino, Italy

[34]Unità di Reumatologia, AOUI, Verona, Italy

[35]Centre for Rheumatology, Royal Free and University College London Medical School, Royal Free Campus, United Kingdom

[36]Institute of Rheumatology, Belgrade, Serbia & Montenegro

[37]Medizinische Universitätsklinik, Abt. II (Onkologie, Hämatologie, Rheumatologie, Immunologie, Pulmonologie), Tübingen, Germany

[38]Rheumatology Granollers General Hospital, Barcelona, Spain

[39]Department of Rheumatology, Marienhospital Stuttgart, Germany

[40]Department of Dermatology, Medical University of Lublin, Poland

[41]Medizinische Universitäts-Poliklinik, Department of Rheumatology, Bonn, Germany

[42]Klinikum der Johann Wolfgang Goethe Universität, Medizinische Klinik III, Rheumatologische Ambulanz, Frankfurt am Main, Germany

[43]Rheumatology Department, Hospitais da Universidade, Coimbra, Portugal

[44]Institute for Prevention, Treatment and Rehabilitation of Rheumatic and Cardiovascular Diseases, Niska Banja, Serbia and Montenegro

[45]Rheumatology Unit, Department of Medicine Chris Hani Haragwanath, Hospital and University of the Witwatersrand, Johannesburg, South Africa

[46]Institute of Rheumatology, Russian Academy of Medical Science, Moscow, Russia

[47]Justus-Liebig University Giessen, Department of Rheumatology and Clinical Immunology, Kerckhoff-Klinik Bad Nauheim, Germany

[48]U.O. Immunologia Clinica - Centro di Riferimento per le Malattie Autoimmuni Sistemiche, Milano, Italy

[49]Department of Rheumatology, University Hospital of Gentofte, Hellerup, Denmark

[50]Servicio de Reumatología, Hospital Ramon Y Cajal, Madrid, Spain

[51]Department of Rheumatology, Rikshospitalet University Hospital, Oslo, Norway

[52]Department of Internal Medicine, Hôpital Claude Huriez, Lille cedex, France

[53]Department of Internal Medicine and Medical Specialities, “Sapienza” University of Rome, Italy

[54]Department of Rheumatology - St. Mary Hospital, Carol Davila, University of Medicine and Pharmacy, Bucharest, Romania

[55]Department of Internal Medicine 3, University Hospital Erlangen, Germany

[56] Division of Rheumatology, Istituto Gaetano Pini, Department of Clinical Sciences & Community Health, University of Milano, Milano, Italy [57]Department of Internal Medicine, Hôpital Cochin, Paris, France

[58]University of Ghent, Department of Rheumatology, Gent, Belgium

[59]U.O. Reumatologia-Università degli Studi di Foggia, Ospedale "Col. D'Avanzo", Foggia, Italy

[60]Dipartimento di Medicina, Ospedale San Gerardo, Monza, Italy

[61]Department of Dermatology and Allergy of the TU Munich, Germany

[62]Department of Rheumatology and Internal Diseases, Wroclaw University of Medicine, Wroclaw, Poland

[63]Endokrinologikum Frankfurt, Germany

[64]Division of Rheumatology, Department of Medicine III/Department of Dermatology, University Medical Center Carl Gustav Carus, Technical University of Dresden, Germany

[65]Catholic University of Leuven, Department of Rheumatology, Leuven, Belgium

[66]University Hospital Centre Zagreb, Division of Clinical Immunology and Rheumatology, Department of Medicine, Zagreb, Croatia

[67]Hospital de Clínicas da Universidade Federal do Paraná, Curitiba - Paraná, Brasil

[68]Municipal Centres of Research in Scleroderma, Hospital "Sacred Trinity", Department of Rheumatology/ Department of Rheumatology, Republican Clinical Hospital, Chisinau, Republic of Moldova

[69]Department of Medicine, Unit of Internal Medicine, Valence cedex 9, France

[70]Dunedin School of Medicine, Dunedin, New Zealand

[71]Department of Medicine, University of Otago, Christchurch, New Zealand

[72]Waikato University Hospital, Rheumatology Unit, Hamilton City, New Zealand

[73]Department of Rheumatology, Bone and Joint Unit, St. Vincent's University Hospital, Dublin, Ireland

[74]Reumatologia e Inmunologia Clinica, Hospital Regional Universitario Jose Ma Cabral y Baez, Clinica Corominas, Santiago, Dominican Republic

[75]Department of Rheumatology, Peking Union Medical College Hospital (West Campus), Chinese Academy of Medical Sciences, Beijing, China

[76]Centro per la Sclerosi Sistemica - Dipartimento di Medicina Clinica, Università La Sapienza, Policlinico Umberto I, Roma, Italy

[77]Clinical Emergency Hospital St. Pantelimon, Bucharest, Romania

[78]U.O. di Reumatologia, A.O.U. Policlinico Vittorio Emanuele, Catania, Italy

[79]Division of Rheumatology & Rehabilitation GR.T.Popa, Center for Biomedical Research, European Center for Translational Research, "GR.T.Popa" University of Medicine and Pharmacy, Rehabilitation Hospital, Iasi, Romania

[80]Department of Rheumatology and Clinical Immunology/Allergology, Inselspital, University of Bern, Switzerland

[81]Hospital Universitario Madrid Norte Sanchinarro, Madrid, Spain

[82]University Hospital of Strasbourg-Department of Rheumatology, Hôpital de Hautepierre, Service de Rhumatologie, Strasbourg Cedex, France

[83]Department of Rheumatology, Tel-Aviv Sourasky Medical Center, Tel-Aviv, Israel

[84]State Research Institute for Innovative Medicine, Vilnius University, Vilnius, Lithuania

[85]Tulane University Lung Center, Tulane/University Medical Center Scleroderma and Sarcoidosis Patient Care and Research Center, New Orleans, USA

[86]Osteoarticular Diseases and Osteoporosis Centre, Pharmacology and Clinical Pharmacological Research Centre, School of medicine - University of Buenos Aires, Rheumatology and Collagenopathies Department, Ramos Mejía Hospital, Buenos Aires, Argentina

[87]USSD Reumatologia, Ospetali Riuniti di Bergamo, Italy

[88]Department of Rheumatology, Clinical Immunology and Allergy, Lausanne, Switzerland

[89]Clinical Immunology and Internal Medicine, National Referral Center for Systemic Autoimmune Diseases, Strasbourg, France

Correspondence and reprints request to:

Oliver Distler, MD, Division of Rheumatology, University Hospital Zurich, Gloriastrasse 25, 8091 Zürich, Switzerland, E-mail: Oliver.Distler@usz.ch, Tel: +41 44 255 2977

Supplementary Methods

Patients and study design

The longitudinally-followed EUSTAR cohort was analysed for this observational study. The whole EUSTAR dataset, consisting of 12,274 patients at the time of the first data export (20.02.2015), was considered.

The following inclusion criteria were used for cohort selection: diagnosis of dcSSc, fulfilment of ACR1980 criteria, mRSS ≥7 at the first visit (baseline) and available data for mRSS at 12±2 months follow-up.

Patients with dcSSc were identified according to LeRoy et al [1] or, in case of missing values for the LeRoy criteria, by the extent of skin involvement at any visit. The minimum

mRSS ≥7 was chosen because it reflects the lowest value classifiable as dcSSc, thus allowing the inclusion of dcSSc patients with less severe to extensive skin fibrosis. The 1 year follow-up has been shown adequate for capturing significant changes in mRSS and is often used in clinical trials in skin fibrosis in SSc.[2]

The clinical data in EUSTAR are prospectively collected in a multicentre approach following a standardized protocol.[3] Regular training courses in skin scoring are organized by EUSTAR and all centres are advised to have the same examiner assessing the skin score in individual patients at follow-up visits.[4] All laboratory investigations including immunological tests are performed according to the local practices of each contributing center, in accordance to international quality standards. Quality indicators for data from the registry include regular external monitoring of large centres, and plausibility checks on key items with written requests to centres for clarification. Ethics approval has been obtained from the respective local ethics committees by all participating EUSTAR centres.

Statistical analysis

The statistical analysis was performed by the biostatistician (NG) using R Version 3.1.0 (packages Hmsic, rms and mice).[5-8]

Definition of variables

The primary endpoint, improvement of skin fibrosis, was defined as a decrease in mRSS of >5 points AND ≥25 % within 1 year. The reduction of >5 points AND ≥25% was chosen in order to capture the minimally clinically important difference.[9] Similarly, progression of skin fibrosis was defined as an increase in mRSS of >5 points AND ≥25 % within one year as used previously.[10]

All standard EUSTAR parameters are described elsewhere.[11,12] The specific variables used for this study are explained below.

Immunosuppressive treatment was defined as explicit documentation of treatment with cyclophosphamide, methotrexate, azathioprine, mycophenolate, d-penicillamine, rituximab, imatinib, TNF inhibitors, and/or prednisone >10mg/day, either at baseline or at follow up visit. This set of medications was chosen because it covers the most relevant and also consistently-reported immunosuppressive agents in the EUSTAR database. Information about immunosuppressive drugs was collected systematically only after 2009, after data collection changed from paper case report forms (CRFs) to electronic CRFs. Patients with mention of receiving at least one of these agents at either baseline or 12-months' follow-up were classified as being treated with immunosuppressives, whereas those with negative inputs for all agents were classified as not having received immunosuppression.

Lung fibrosis was defined as fibrosis on HRCT and, additionally, as fibrosis on chest X-ray.

Selection of parameters for multivariable analysis

Parameters for multivariable analysis were selected exclusively based on expert opinion. Scleroderma experts (CM, OK, OD, YA, RD) were asked to suggest parameters that could be important for skin improvement, taking into account face validity, clinical and scientific reasoning. All suggestions were gathered as received, adding to a total of 19 parameters. Out of these, to allow a trustworthy imputation, only the parameters with >50% valid values were further considered for the analysis. This was acknowledged as a limitation. Regressors were no more likely than non-regressors to have missing values on any of the variables. An overview on all the suggested parameters is presented in Table S1, whereas the ones finally selected for the analysis are also shown in Table 2, main manuscript.

Table S1. Overview on missing data for the candidate predictors of skin improvement suggested by the scleroderma experts

Variable	Missing	
	N	%
Baseline mRSS	0	0.0
Disease duration	65	7.1
ANA positive	11	1.2
Anti Scl70 positive	33	3.6
Joint contractures	3	0.3
Tendon friction rubs	5	0.5
Proteinuria	32	3.5
Conduction blocks	42	4.6
Abnormal diastolic function	55	6.0
Fibrosis on chest X-ray	70	7.6
DLCO≥70%	298	32.4
Immunosuppression	483	52.6
Active digital ulcers	525	57.1
Scleredema (puffy fingers)	529	57.6
CRP elevation	545	59.3
ESR<25mm/1h	551	60.0
LVEF <45%	601	65.4
Lung fibrosis on HRCT	632	68.8
Anti-RNA polymerase III positive	704	76.6

Abbreviations: mRSS: modified Rodnan skin score; ANA: antinuclear antibodies; X-ray: radiography; DLCO: diffusion capacity of the lung for carbon monoxide; CRP: C-reactive

protein; ESR: erythrocyte sedimentation rate; LVEF: left ventricular ejection fraction; HRCT: high resolution computed tomography of the chest.

A comparison of the selected candidate variables between the patients who met all inclusion criteria (diagnosis of dcSSc, fulfilment of ACR1980 criteria, mRSS ≥7 at the first visit and available data for mRSS at 12±2 months follow-up) and were, therefore, included into the analysis ("selected") and those patients who could not be analyzed because they did not have a second follow-up visit within the required timeframe ("not selected") did not reveal significant differences between these two groups (Table S2).

Table S2. Frequencies of the candidate predictors of skin improvement in the selected cohort (patients with both baseline and 1-year follow up visits), compared to the non-selected patients with dcSSc but not meeting the two required consecutive visits.

Parameters	Selected (n=919)	Not selected (n=2310)
ANA positive	94.6%	93.3%
Scl70 positive	59.1%	58.3%
Tendon friction rubs	20.2%	18.2%
Proteinuria	8.3%	8.0%
Conductions blocks	12.4%	13.4%
Abnormal diastolic function	19.1%	18.7%
Lung fibrosis on X-ray	45.8%	49.2%
DLCO≥70%	42.5%	38.4%
Disease duration (months)	Median:42.5	Median:49
Baseline mRSS	Median:16	Median:18

Abbreviations: mRSS: modified Rodnan skin score; ANA: antinuclear antibodies; X-ray:

radiography; DLCO: diffusion capacity of the lung for carbon monoxide; CRP: C-reactive protein; ESR: erythrocyte sedimentation rate; LVEF: left ventricular ejection fraction; HRCT: high resolution computed tomography of the chest.

Imputation of missing data and predictive modelling

Single conditional mean imputation and validation through Bootstrap

A logistic regression model was fit after single conditional mean imputation of missing values. Multiple imputation is clearly superior to single conditional mean imputation, however, it is not possible to validate models with multiply imputed data. Therefore, the models were validated after single conditional mean imputation using the Bootstrap methods with 100 repetitions.

The method of single conditional mean imputation fills in missings with predicted values from using the multivariable imputation model based on non-missing data. Subsequently, a model was run with all potential predictors. Baseline mRSS was centered at 7 points as all included patients had mRSS ≥7. The linearity assumption was relaxed for baseline mRSS and disease duration by including restricted spline functions with 4 knots. The interaction between disease duration and baseline mRSS was also tested, but proved to be insignificant, meaning that the effect of baseline mRSS on regression of dSSc did not depend on values of disease duration.

Multiple imputation

Multiple imputation was used to fit the full und reduced model and to get standard errors. Missing values were multiply imputed with help of the R package mice. For the imputation model, all variables from the full model were included, i.e., all 11 variables as well as the dependent variable “regression of mRSS”. Moreover, the time of the first visit (before 2009 vs. 2009 or later) was also included as it was strongly related to nonresponse: data collection was changed to an online version between 2008 and 2009 and some of the items were not

collected until after 2009. In addition, variables with an absolute correlation with the target variables of at least 0.2 were included. Only variables with a proportion of usable cases (cases with missing data on the target variable that had observed values on the predictor) of at least 25% were retained in the imputation model as to many missing cases on the same cases for both the target and the predictor variable would not contain much information to impute the target variable. The order in which variables should be imputed was defined according to their number of missing cases. Depending on the scale of the target variable, multiple imputation was performed using either predictive mean matching (pmm) or logistic regression (logreg). Ten imputed data sets were generated. Imputation was assessed via density plots for plausibility, i.e. whether imputed data were possible and close to the observed data.

Supplementary results

Single mean imputation, development and validation of the prediction model

A model was run with all the selected potential predictors (Table 2). The Wald statistics indicated that disease duration could be modelled linearly (Table S3). Baseline mRSS, however, did not behave linearly. Therefore, a quadratic term was included for baseline mRSS. As the effects were clearly insignificant (P>0.7) for joint contractures and DLCO≥70%, these effects were excluded from any future models (Table S3 and S4).

Table S3. Wald statistics for the regression model for skin improvement at one year after single conditional mean imputation

Factor	Chi-square	Degrees of freedom	P-value
ANA positive	1.28	1	0.259
Anti Scl70 positive	3.96	1	0.047
Joint contractures	0.01	1	0.919
Tendon friction rubs	7.74	1	0.030
Proteinuria	0.62	1	0.430
Conduction blocks	1.01	1	0.316
Abnormal diastolic function	0.96	1	0.323
Fibrosis on chest X-ray	1.19	1	0.276
DLCO≥70%	0.14	1	0.701
Baseline mRSS	70.08	3	<0.001
nonlinear	9.30	2	0.001
Disease duration	1.21	3	0.752

nonlinear	0.45	2	0.799
Total nonlinear	9.55	4	0.049
Total	89.38	15	<0.0001

Abbreviations: mRSS: modified Rodnan skin score; ANA: antinuclear antibodies; X-ray: radiography; DLCO: diffusion capacity of the lung for carbon monoxide; CRP: C-reactive protein; ESR: erythrocyte sedimentation rate; LVEF: left ventricular ejection fraction; HRCT: high resolution computed tomography of the chest.

The full regression model for prediction of skin improvement is shown in **Error! Reference source not found.**.

Table S4. Full prediction model for skin improvement after single conditional mean imputation

Variable	Coefficient	Standard error	Odds ratio	P-value
ANA positive	-0.400	0.34	0.67	0.229
**Anti Scl70 positive**	**-0.358**	**0.18**	**0.70**	**0.044**
**Tendon friction rubs**	**-0.480**	**0.22**	**0.62**	**0.026**
Proteinuria	0.227	0.29	1.25	0.431
Conduction blocks	0.220	0.24	1.25	0.365
Abnormal diastolic function	0.214	0.21	1.24	0.317
Fibrosis on chest X-ray	0.181	0.18	1.20	0.304
**Baseline mRSS**	**0.202**	**0.04**	**1.22**	**<0.0001**
**Baseline mRSS^2**	**-0.003**	**0.00**	**1.00**	**0.006**
Disease duration	-0.001	0.00	1.00	0.369
Intercept	-3.373	0.57	0.03	<0.0001

Abbreviations: mRSS: modified Rodnan skin score; ANA: antinuclear antibodies; X-ray:

radiography; DLCO: diffusion capacity of the lung for carbon monoxide; CRP: C-reactive protein; ESR: erythrocyte sedimentation rate; LVEF: left ventricular ejection fraction; HRCT: high resolution computed tomography of the chest.

For the validation, the Bootstrap method was used. Table S5 shows the performance of the model. Discrimination refers to the ability of the model to separate subjects with and without the outcome. The C-index as a measure to estimate discrimination was 0.7231 for the full model, which was reduced to 0.7071 at validation. Calibration refers to the agreement between actual and predicted probabilities. The slope shrinkage factor was 0.9117 and the maximum absolute error in predicted probability was 0.0347. Thus, there was some overfitting present. Moreover, the model could only explain 13.4% of the variation at validation.

Table S5. Performance of the prediction model for skin improvement before and at validation

**Performance measure**	**Full model**	**Validation full model**
R^2	0.1615	0.1342
C-index (AUC)	0.7231	0.7071
Calibration slope	1.0000	0.9117
E_{max}	0.0000	0.0347

R^2: R-squared, the percentage of the response variable variation that is explained by a linear model. AUC: Area under the curve; E_{max}: maximum absolute error.

Baseline mRSS as a predictor of the change in skin score at one year

In the current cohort, 95/919 (10%) dcSSc patients who showed skin progression within one year had lower baseline mRSS ($p<0.001$). Baseline mRSS is thus a predictor of change in skin score after 1 year, patients with lower skin scores being prone to progress and those with higher skin scores to improve within the next 12 months (Figure S1).

Figure S1. Baseline mRSS in patients with and without skin regression. Patients with skin regression (black bars) have higher baseline mRSS values relative to patients without skin regression (grey bars).

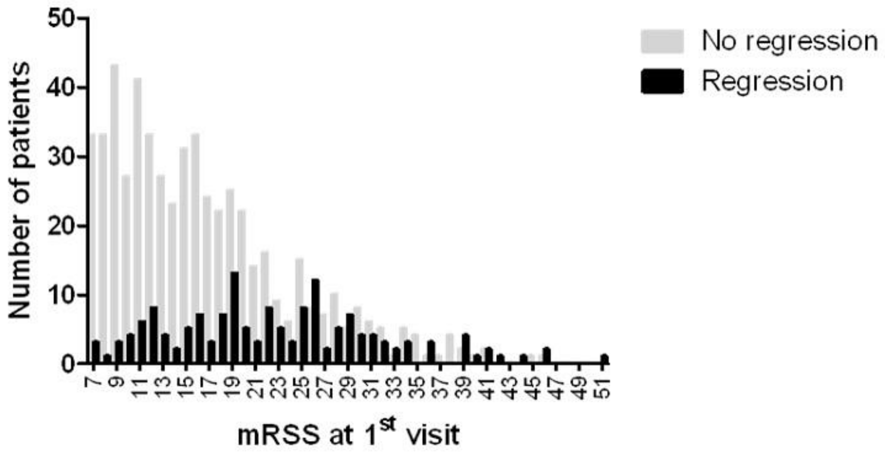

Supplementary references:

1.LeRoy EC, Black C, Fleischmajer R, et al. Scleroderma (systemic sclerosis): classification, subsets and pathogenesis. J Rheumatol. 1988 Feb; 15(2):202-5.

2.Wiese AB, Berrocal VJ, Furst DE, et al. Correlates and responsiveness to change of measures of skin and musculoskeletal disease in early diffuse systemic sclerosis. Arthritis Care Res (Hoboken). 2014 Nov; 66(11):1731-9.

3.Muller-Ladner U, Tyndall A, Czirjak L, et al. Ten years EULAR Scleroderma Research and Trials (EUSTAR): what has been achieved? Ann Rheum Dis. 2014 Feb; 73(2):324-7.

4.Czirjak L, Nagy Z, Aringer M, et al. The EUSTAR model for teaching and implementing the modified Rodnan skin score in systemic sclerosis. Ann Rheum Dis. 2007 Jul; 66(7):966-9.

5. R Core Team (2014). R: A language and environment for statistical computing. R Foundation for Statistical Computing, Vienna, Austria. ULR http://www.R-project.org/

6. Stef van Buuren, Karin Groothuis-Oudshoorn (2011). mice: Multivariate Imputation by Chained Equations in R. Journal of Statistical Software, 45(3), 1-67. URL http://www.jstatsoft.org/v45/i03/.

7. Frank E Harrell Jr, with contributions from Charles Dupont and many others (2015). Hmisc: Harrell Miscellaneous. R package version 3.16-0. URL http://CRAN.R-project.org/package=Hmisc

8. Frank E Harrell Jr (2015). Rms: Regression Modeling Strategies. R package version 4.3-1. ULR http://CRAN.R-project.org/package=rms

9. Khanna D, Furst DE, Hays RD, et al. Minimally important difference in diffuse systemic sclerosis: results from the D-penicillamine study. Annals of the rheumatic diseases. 2006 Oct; 65(10):1325-9.10. Walker UA, Tyndall A, Czirjak L, et al. Clinical risk assessment of organ manifestations in systemic sclerosis: a report from the EULAR Scleroderma Trials And Research group database. Ann Rheum Dis. 2007 Jun; 66(6):754-63.

11.Galluccio F, Walker UA, Nihtyanova S, et al. Registries in systemic sclerosis: a worldwide experience. Rheumatology (Oxford). 2011 Jan; 50(1):60-8.
